# Efficacy of tocilizumab therapy in a patient with severe pancytopenia associated with a STAT3 gain-of-function mutation

**DOI:** 10.1186/s12865-021-00411-1

**Published:** 2021-03-17

**Authors:** Wenjie Wang, Luyao Liu, Xiaoying Hui, Ying Wang, Wenjing Ying, Qinhua Zhou, Jia Hou, Mi Yang, Bijun Sun, Jinqiao Sun, Xiaochuan Wang

**Affiliations:** Department of Clinical Immunology, Children’s Hospital of Fudan University, National Children’s Medical Center, Shanghai, 201102 China

**Keywords:** STAT3 transcription factor, Tocilizumab, Immune Dysregulation, Gain-of-function mutations

## Abstract

**Background:**

We aimed to report the clinical characteristics, immunological features, and treatment of one patient with a de novo STAT3 gain-of-function mutation identified by next generation sequencing. We investigated the efficacy of tocilizumab therapy in immune dysregulation diseases caused by STAT3 mutation.

**Results:**

The patient was a 16-year-old girl. She presented with recurrent respiratory infections and chronic diarrhea after birth. She had life-threatening autoimmune pancytopenia at 14 years old. After receiving glucocorticoid therapy, she developed diabetes. However, her pancytopenia relapsed when the glucocorticoid was tapered. Next-generation sequencing showed a de novo heterozygous mutation in the STAT3 gene, c.1261G > A (p. G421R), which was previously described as a gain-of-function mutation. After tocilizumab therapy, her pancytopenia fully resolved, and insulin and glucocorticoid therapies were gradually discontinued within 12 months. She had lymphopenia and an inverted CD4/CD8 ratio before therapy. Lymphocyte subpopulation analysis indicated an expansion of effector memory CD4+, effector memory CD8+ and central memory CD4+ T cells. The proportions of memory B cells and naive CD4+ T cells were decreased, and the proportion of naïve B cells was increased. None of the abnormal lymphocytic changes improved significantly. STAT3 GOF mutations were identified by next gene sequencing in those with early-onset multi-organ autoimmunity. Including our patient, 13 patients with STAT3 GOF mutations received targeted treatment. Twelve of them were treated with tocilizumab alone or combination tocilizumab with JAK inhibitor, and ten patients improved.

**Conclusions:**

Gene sequencing should be performed for patients with early-onset refractory or multiorgan immune dysregulation diseases. Targeted drugs can effectively improve the clinical problems associated with STAT3 gain-of-function mutations, while nontargeted immunosuppressive therapy is usually insufficient.

**Supplementary Information:**

The online version contains supplementary material available at 10.1186/s12865-021-00411-1.

## Background

Signal Transduction and Activator of Transcription 3 (STAT3), one of the seven members of the STAT family of transcription factors, helps transmit key cytokine signals from cell membrane receptors to the nucleus using Janus kinases (JAKs). STATs are involved in multiple processes, including early development, cellular proliferation, survival, and differentiation. Germline STAT3 gene loss-of-function (LOF) mutations cause hyper IgE syndrome, which often manifests as special facial features, recurrent infections, eczema, and joint hyperextension [1]. STAT3 gain-of-function (GOF) mutations were first reported in 2014 [2–8]. In the phenotypical classification of inborn errors in human immunity by the International Union of Immunological Societies (IUIS) in the 2019 update [9], diseases of immune dysregulation were divided into two categories: a) hematopoietic lymphohistiocytosis syndrome (HLH) and Epstein-Barr virus (EBV) susceptibility and b) syndromes with autoimmunity and others. GOF mutation of the germline STAT3 gene is one of the regulatory T cell defects accompanied by autoimmune manifestations. These mutations cause early-onset lymphoproliferation with lymphadenopathy, hepatosplenomegaly and multiorgan autoimmunity, including cytopenia, hepatitis, inflammatory lung disease, enteropathy, and diabetes mellitus.

Patients with STAT3 GOF were treated with regular immunosuppressive treatments before the results of gene sequencing which always couldn’t control all the clinical manifestations [10–14]. Functional analyses of STAT3 GOF mutations demonstrated increased transcriptional activity of STAT3 in unstimulated and/or stimulated cells in vitro [3]. Because IL-6 is one of the primary cytokines that utilizes STAT3 for signal transduction, IL-6 receptor (IL-6R) antagonist therapy currently have been reported to be effective in these patients [4, 5, 10–13]. Here, we reviewed the clinical manifestations and immunological features of one patient with a STAT3 GOF mutation. Moreover, we aimed to investigate the efficacy of tocilizumab therapy.

## Results

### Case review

**Fig. 1:**
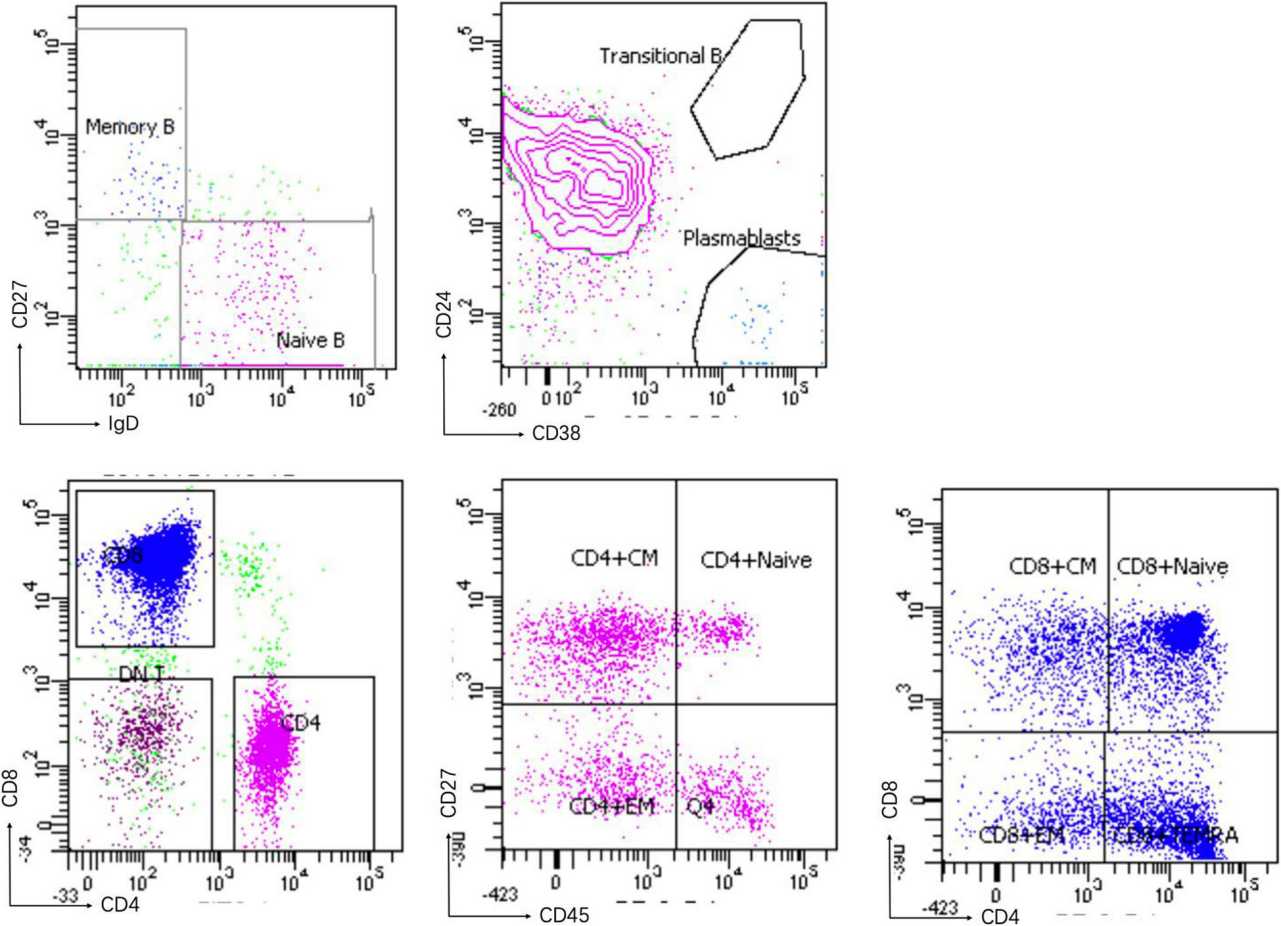
Lymphocyte subpopulaltions of our patient. A:CD19 + IgD + CD27– naïve B (94.2%), CD19 + IgD–CD27+ memory B (1.9%); B:CD38 + CD24+ transitional B (0%), CD38 + CD24– plasmablast B (1.7%); C: CD3 + CD4+ T (24.4%); CD3 + CD8+ T (66.3%); CD3 + CD4–CD8– double negative T (7.2%); D:subpopulaltions of CD4+ T cells: CD3 + CD4 + CD45RA + CD27+ naïve T (11%), CD3 + CD4 + CD45RO + CD27+ central memory T (49.6%), CD3 + CD4 + CD45RO + CD27– effector memory T (23.8%); E:subpopulaltions of CD8 + T cells: CD3 + CD8 + CD45RA + CD27+ naïve T (48.1%), CD3 + CD8 + CD45RO + CD27+ central memory T (13.7%), CD3 + CD8 + CD45RO + CD27– effect memory T (9.3%), CD3 + CD8 + CD45RA + CD27– TEMRA T (28.9%)

A 16-year-old girl born to nonconsanguineous Chinese parents presented with recurrent respiratory infections after birth and suffered at least 1–2 pneumonia events per year. She had chronic diarrhea from 3 months old. When she was 12 years old, she developed cervical adenopathy, alopecia, and bilateral lower limb weakness. She had life-threatening autoimmune pancytopenia with a positive Coomb’s test at the age of 14. She also presented with growth retardation and hepatosplenomegaly. Laboratory data showed that her peripheral blood leukocyte count was (0.90–1.36) × 10^9^/L, her hemoglobin level was 87–106 g/L and her platelet count was (32–51) × 10^9^/L. Her antinuclear antibody (ANA), extractable nuclear antibody (ENA) spectrum and anticardiolipin antibody detection were negative. Complement C3 and C4 were normal. She had lymphopenia and an inverted CD4/CD8 ratio before therapy, and her serum immunoglobulin G concentration was slightly higher than the normal range. Lymphocyte subpopulation analysis indicated expansion of effector memory (EM) CD4+ T, EM CD8+ T and central memory (CM) CD4+ T cells. The proportions of memory B cells and naive CD4+ T cells were decreased, and the proportion of naïve B cells was increased (Fig. 1). Magnetic resonance imaging of the lower limbs revealed abnormal signals in the muscles and fluid accumulation in the intermuscular space, which indicated inflammatory changes.

### Gene analysis

**Fig. 2:**
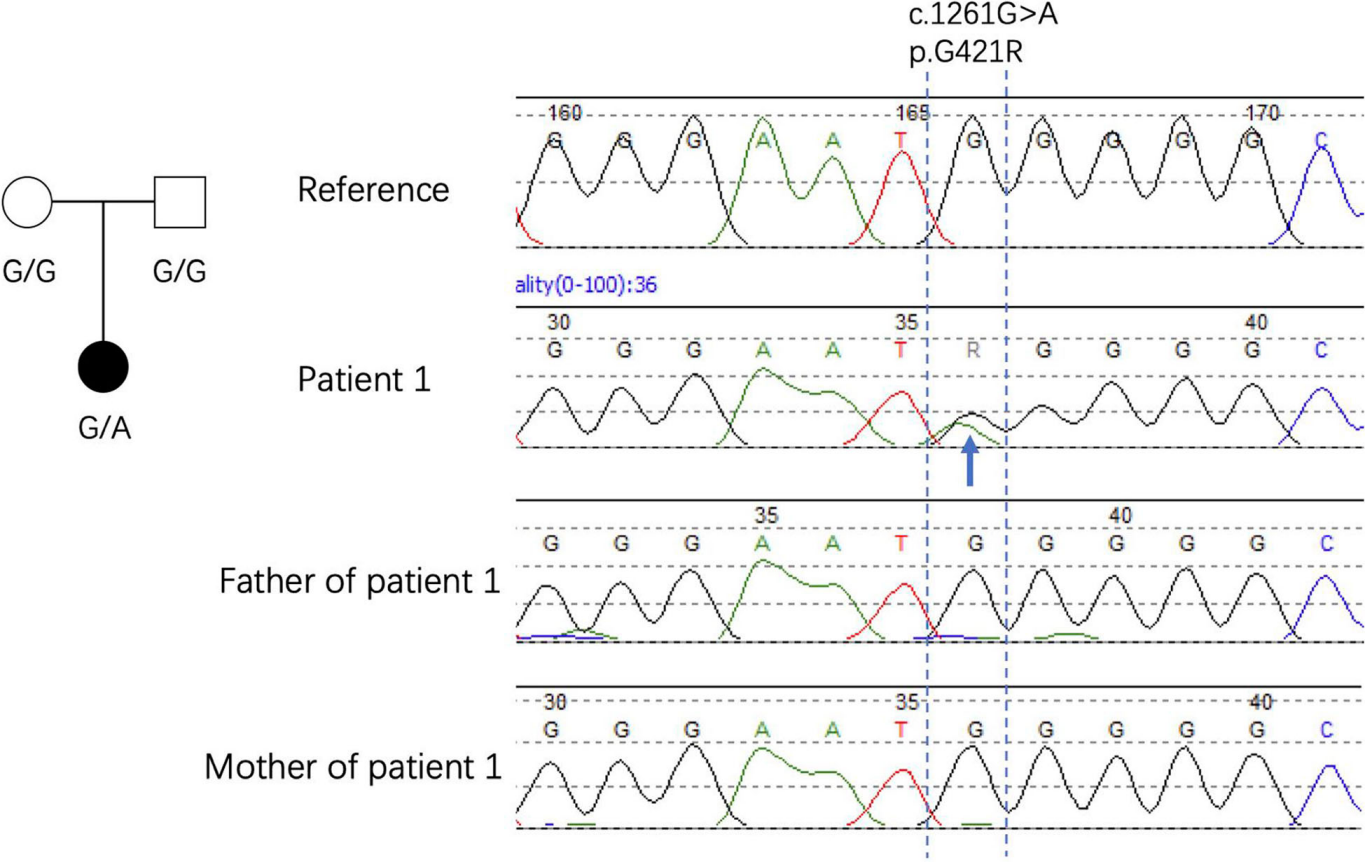
Sanger sequence results of our patient and her parents

Next-gene sequencing (NGS) revealed that she had a heterozygous de novo mutation in the STAT3 gene, while her parents were normal (Fig. 2). The missense mutation in the DNA binding domain (c.1261G > A, p. G421R) was described as a gain-of-function mutation in previous studies [2].

### Treatment and follow-up

Before the gene sequencing results were available, the patient accepted intravenous methylprednisolone (1.6 mg/kg/day) for autoimmune pancytopenia. She was changed to the same dose of oral glucocorticoid tablet after Coomb’s test turned negative and pancytopenia improved. But the pancytopenia relapsed several times once the glucocorticoid dose was less than 2 mg/kg/day. Meanwhile, she developed diabetes and worse weakness of lower limbs during the glucocorticoid treatment. The fasting plasma glucose was 7.2 mmol/L, the glycated hemoglobin (Hb1c) was 5.8%, and all the insulin autoantibodies were negative. The oral glucose tolerance test showed increased C peptide and plasma glucose after 30 min, 60 min, 120 min, and 180 min of oral glucose, while plasma insulin level didn’t increase (supplement Table 2). So, we diagnosed diabetes and considered it as a side effect of glucocorticoid therapy. The blood biochemistry results were normal before and after treatment, including creatine kinase (CK), serum electrolytes, transaminase, and creatinine. The diagnosis of myositis was based on MRI abnormal signals in muscle and cutting movement assessment score (CMAS) was 15 of 52.

**Fig. 3:**
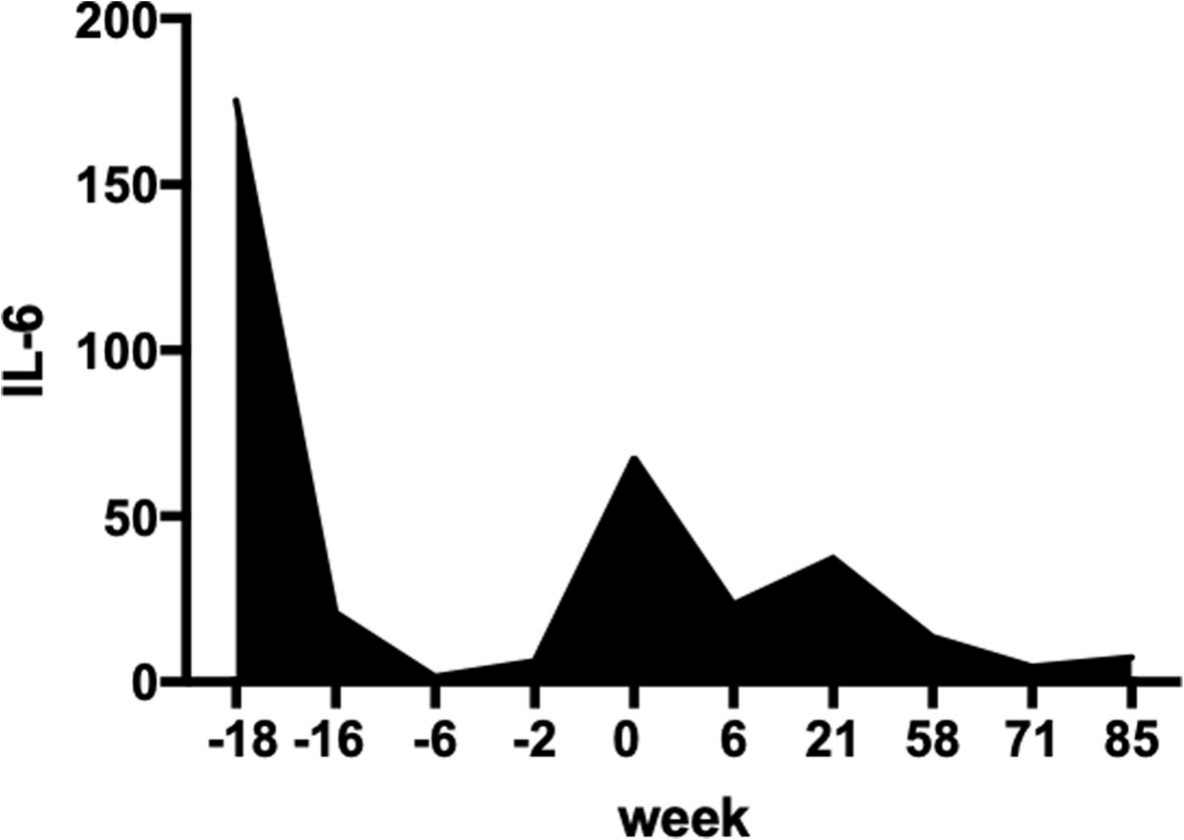
Serum IL-6 before and after tocilizumab of our patient. She accepted glucocorticoid first from week − 18 to week 0

After obtaining informed consent from her parents, we administered IL-6R antagonist treatment (tocilizumab) at a dosage of 8 mg/kg intravenous infusion every 2 weeks. After 2 months of tocilizumab treatment, the patient’s pancytopenia fully resolved. She discontinued insulin injection after 4 months of tocilizumab therapy, and glucocorticoid therapy was gradually discontinued within 12 months. Her serum IL-6 levels increased before glucocorticoid therapy and improved for a while but fluctuated after glucocorticoid tapering and gradually decreased to normal after tocilizumab therapy (Fig. 3). She underwent intravenous immunoglobulin (IVIG) replacement after 6 months because of hypogammaglobulinemia. Beginning in the second year of therapy, she extended the interval of tocilizumab to every 4–6 weeks (240 mg/dose, after her weight was above 30 kg), with a good response evidenced by a normal hemoglobin level and negative Coomb’s test. Lymphadenopathy and hepatosplenomegaly returned to mild enlargement (Table 1).

**Table 1:** Clinical features and immunotype of our patient with a STAT3 GOF mutation

tocilizumab therapy	before	after
lymphadenopathy	yes	no
hepatosplenomegaly	yes (severe)	mild
autoimmune pancytopenia	yes	no
myositis	worse	better
alopecia	yes	better
WBC (×10^9^/L)	0.90–1.36^#^ 0.9–6.8^##^	4.7–8.3^###^
HB g/L	87–106^#^ 46–139^##^	112–139^###^
PLT (×10^9^/L)	32–51^#^ 32–123^##^	107–178^###^
IgM	1.84	0.62
IgA	1.3	0.25
IgG	14.5	6.6 ^a^
IgE	< 2	< 2
CD3	89.13% (607)	82.87% (786)
CD4	32.41% (221)	30.61% (290)
CD8	51.17% (349)	48.70% (462)
CD4/CD8	0.63	0.63
CD19	8.74% (60)	9.08% (31)
NK	1.49% (10)	7.28% (69)

* with monthly IVIG replacement

# before glucocorticoid treatment, ## after glucocorticoid treatment;

### after 1 month of tocilizumab therapy

We regularly followed up for infection indicators during targeted therapy. She had cervical lymphadenitis and *Candida albicans* vaginitis once during 2 years of therapy. Her EBV-DNA load of whole leukocytes in the peripheral blood was higher than the level at the beginning, changing from 1.29 × 10^4^ copies/ml to 3.58 × 10^6^ copies/ml. In addition to treatment with targeted drugs, she accepted oral itraconazole and acyclovir tablets.

**Fig. 4:**
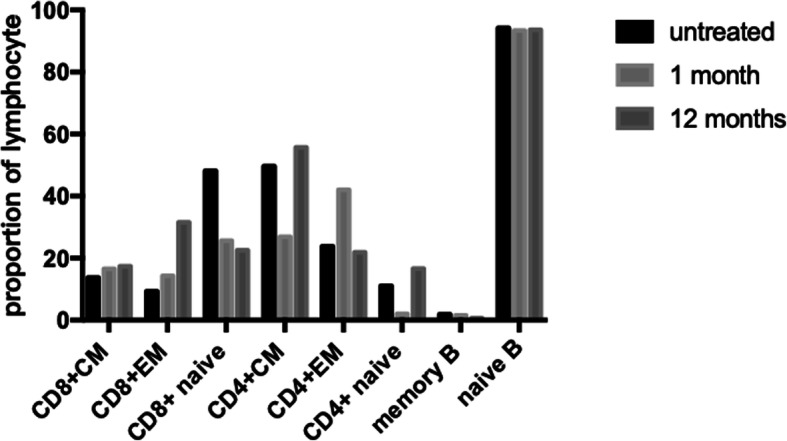
Subpopulation of T/B lymphocytes of our patient before and 1 month, 12 months after tocilizumab

Her immune phenotypes after tocilizumab showed hypogammaglobulinemia after 6 months of therapy. All the abnormal lymphocytic changes (expansion of EM CD4+ T, EM CD8+ T and CM CD4+ T cells; decreased memory B cell and naive CD4+ T cell levels; and an increased naïve B cell level) did not improve significantly (Fig. 4).

## Discussion

**Fig. 5:**
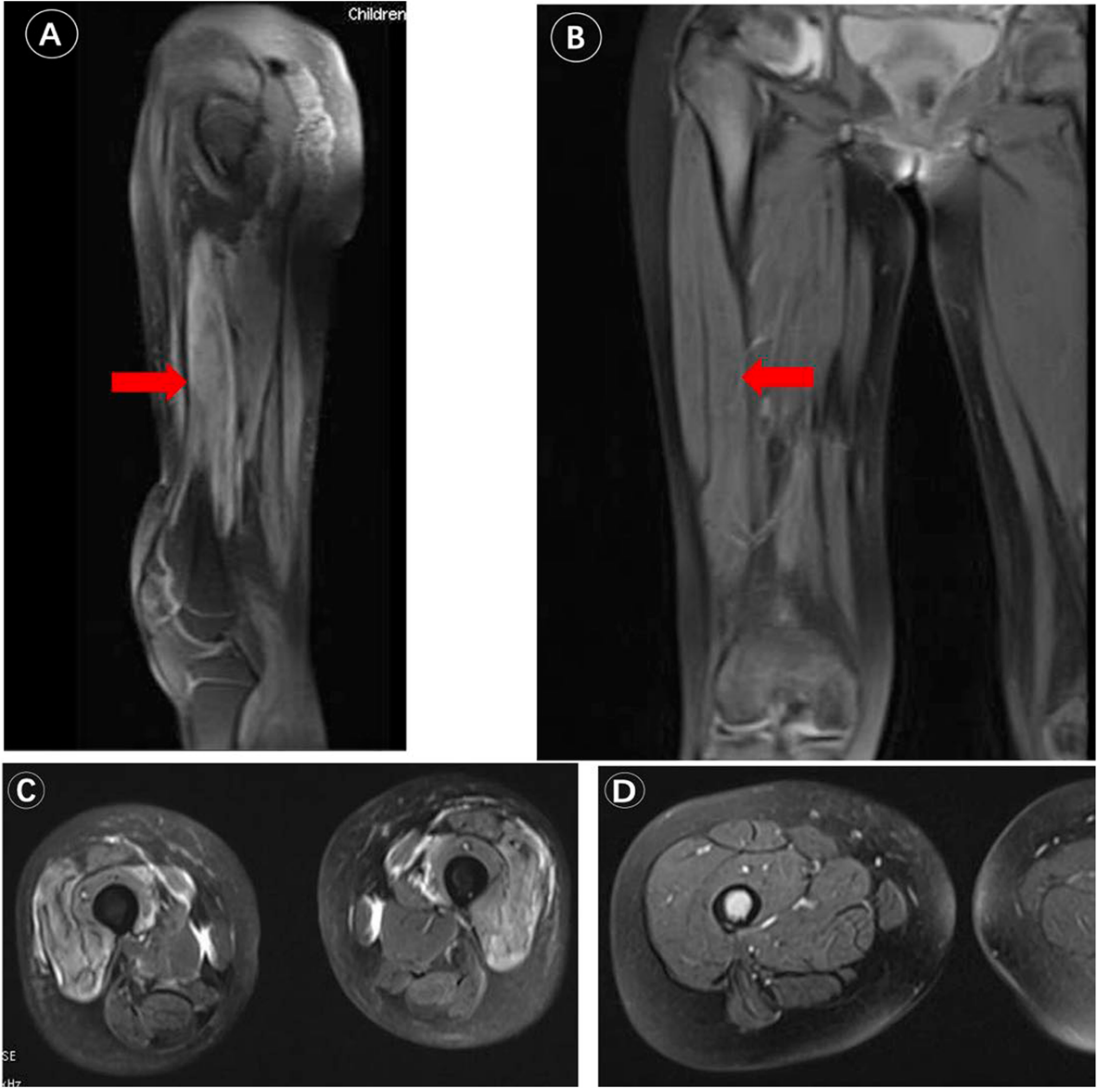
Magnetic resonance imaging of left lower limbs of patient 1. **a** and **c** revealed abnormal signals in muscle and fluid accumulation in the intermuscular space considering inflammatory changes. **b** and **d** revealed abnormal changes recovered after tocilizumab

STAT3 GOF mutations were reported in patients with multiple early-onset autoimmune manifestations by NGS [3]. Fabre et al. summarized the clinical features of 42 patients, and the most common manifestations were autoimmune cytopenia, lymphoproliferation, enteropathy, interstitial lung disease, thyroiditis, diabetes, and growth retardation; some of the patients had recurrent or opportunistic infections [14]. The onset age and major symptoms of our patient were similar to those of other patients reported previously [2, 3]. She developed diabetes during glucocorticoid therapy with normal glycated hemoglobin levels, which is different from the early-onset type 1 diabetes reported in the literature. Her Hb1c, insulin autoantibodies and oral glucose tolerance test indicated her diabetes was a side effect of glucocorticoid therapy. We also found that her bilateral lower limb weakness was related to immune myositis on MRI. The abnormal changes in the muscles improved after tocilizumab treatment (Fig. 5).

According the functions of immune system, infection tendency, autoimmunity and extremely abnormal immune reactions are common manifestations of primary immunodeficiencies. So, we did NGS at the beginning of diagnose considering a genetic defect may exist. STAT3 GOF mutations were identified by gene sequencing in those with early-onset multi-organ autoimmunity.

Activation of STAT3 leads to the production of downstream cytokines, including glycoprotein 130 (a component of IL-6 and IL-27), common gamma chain cytokines, IL-10 family cytokines and IL-23. After cytokines bind to their receptors, one of four Janus kinases (JAKs) is activated and phosphorylates the cytokine receptor. STAT molecules are recruited to the phosphorylated receptor and then translocate to the nucleus, altering gene expression [15]. The proportions of regulatory T cells are reduced, while IL-17 levels are increased in patients with STAT3 GOF mutations [3, 8]. In vitro, IL-6R antagonists can reduce IL-17 secretion and improve immune dysregulation by increasing regulatory T cell differentiation [4]. Therefore, IL-6R antagonists and JAK inhibitors can inhibit STAT3 signaling pathway overactivation and be used to treat patients with STAT3 GOF mutations.

Twelve patients with STAT3 GOF mutations received targeted treatment in 5 reports [4, 5, 9–11]. Three (including our patient, P1) of the 13 patients were treated with tocilizumab alone, and all three improved (P1, P2, and P3); 1 patient improved with JAK inhibitor treatment alone (P7); the remaining 9 patients were treated successively or simultaneously with tocilizumab and JAK inhibitors because of disease severity or incomplete remission. Combination treatment could take the following factors into consideration: enteropathy (4/9), interstitial pulmonary disease (4/9), arthritis (2/9), hepatitis (3/9), splenomegaly (1/9), and cytopenia (2/9). Seven of the nine patients who received combined treatment achieved remission (Table 2). STAT3 GOF mutation patients manifesting with multiple clinical symptoms might need combination therapy if the tocilizumab or JAK inhibitors alone couldn’t control the disease. The outcomes of targeted therapy are summarized in Table 3. The symptoms of arthritis, cytopenia, hepatosplenomegaly and hepatitis were all alleviated. Three of the 13 patients died. P10 died of infection after transplant, while recurrent HLH was resolved by targeted treatment. P9 died of sepsis and diffuse intravascular coagulation. P13 died of respiratory failure. Considering cytopenia as the common adverse reaction to JAK inhibitors, two patients with the G421R mutant who recovered with tocilizumab alone have been reported in the literature [2, 4]. Therefore, we chose tocilizumab as the first targeted treatment for our patient. After 1 year of treatment, her whole blood cells stayed in the normal range with no serious infections. However, the EBV-DNA titer of peripheral blood leukocytes was higher than that at the beginning. This means that latency and active infections need to be monitored before and after targeted therapy.

**Table 2:** Summary of targeted therapy in all the reported patients with a STAT3 GOF mutation

Patient no.	1	2	3	4	5	6	7	8	9	10	11	12	13
a. sex	female	male	male	female	female	female		male	female	male	female	male	male
b. onset age	3 mo	4 yr	6 mo	5 mo	1 yr	1 yr	1 mo	7 yr	3 yr	7 mo			7 yr
c. age	16 yr	10 yr	38 yr	19 yr	9 yr	21 yr	20 yr	8 yr	14 yr	3 yr	8 yr	13 yr	15 yr
d. infections	Y		Y	Y				Y	Y				
e. cytopenia	Y	Y		Y				Y	Y		Y	Y	Y
f. enteropathy			Y	Y	Y	Y		Y	Y	Y	Y		Y
g. hepatitis		Y							Y		Y	Y	Y
h. endocrine disease	Y			Y	Y				Y				Y
i. arthritis		Y			Y	Y	Y					Y	
j. lymphadenopathy	Y	Y											Y
k. hepatosplenomegaly	Y	Y	Y		Y			Y	Y	Y	Y	Y	Y
l. interstitial lung disease					Y	Y	Y				Y		Y
m. short stature	Y	Y	Y	Y	Y	Y		Y	Y	Y	Y	Y	Y
n. reason of for targeted therapy	pancytopenia,	arthritis	enteropathy,	colitis	interstitial lung disease	arthritis,	interstitial lung disease	hepatosplenomegaly,	hepatotitis,	enteropathy,	interstitial lung disease	arthritis	enteropathy,
	hepatosplenomegaly		dermopathy			interstitial lung disease		anemia	enteropathy HLH				interstitial lung disease
o. tocilizumab alone	✓	✓	✓										
p. combination of tocilizumab and a JAK inhibitor				✓	✓	✓		✓	✓	✓	✓	✓	✓
q. JAK inhibitor alone							✓						
r. efficiency of targeted therapy	yes	yes	yes	yes	yes	yes	yes	yes	no	yes	yes	yes	no
s. outcome	alive	alive	alive	alive	alive	alive	alive	alive	dead	dead	dead	alive	dead
t. mutantmutation in of STAT3	G421R	G421R	G421R	N401D	E415L	V393A	M329K	R152W	F174S	E286G	Q344H	G421R	P715L
u. reference	our report	[2]	[4]	[13]	[11]	[12]	[11]	[10]	[10]	[10]	[10]	[10]	[10]

Y represent YES, and slash indicate the parameter was not mentioned in the reference

p: combination of tocilizumab and JAK inhibitors

Patients 4, 5, 6, 9, 11 and 12 were treated with tocilizumab first, and then a JAK inhibitor was added later. Patients 8 and 10 were treated with two targeted drugs simultaneously. Patient 14 was treated with a JAK inhibitor first and then changed to tocilizumab

**Table 3:** Effect summary for targeted therapy

Cause of targeted therapy	Case number	Remission number	No response	Proportion of remission
enteropathy	5	3	2	60%
ILD	5	4	1	80%
arthritis	3	3	0	100%
cytopenia	3	3	0	100%
hepatosplenomegaly	2	2	0	100%
dermopathy	2	1	1	50%
hepatitis/liver failure	1	0	1	0%

The immunological features of STAT3 GOF mutations included hypogammaglobulinemia, increased Th17 cells and decreased Treg cells, which plays a critical role in the development of autoimmunity. While some case reports showed normal Th17 cells or Treg cells in the symptomatic STAT3 GOF patients [8]. There is no immunological index for starting and evaluating the efficacy of targeted therapy. The 12 patients reported above had no immunophenotypic changes with targeted treatment. One patient showed an improvement in the proportion of regulatory T cells after tocilizumab treatment [4]. We evaluated lymphocyte subpopulations before and after treatment. There were no significant improvements in those abnormal changes in our patient after 1 year of treatment. It cannot be ruled out that the immune phenotype changes later than clinical improvement. The relationship between immunological characteristics and mechanism of disease needs further research. The patient still requires monthly IVIG replacement with targeted therapy. Consistent with other reports [2, 16], she had hypogammaglobulinemia and a low proportion of memory B cells. However, the IgG level was higher than normal during the course of autoimmune pancytopenia due to the presence of autoantibodies. Therefore, her hypogammaglobulinemia was consistent with the reduced proportion of memory B cells regardless of the side effects of tocilizumab. The pathogenesis of the abnormal changes leading to reduced unswitched memory B cells and hypogammaglobulinemia require further study.

It has been reported that 5 patients underwent hematopoietic stem cell transplantation [2, 9, 17, 18], of whom four died due to complications, such as infection. The remaining patient was successfully transplanted and lived well. It is known from the reported cases that targeted therapy can effectively control and alleviate life-threatening autoimmune manifestations and lymphoproliferative disorders. The longest follow-up time for targeted therapy is 3.5 years [9]. Our patient was followed up for 2 years, and she had no obvious serious infections or adverse reactions. Aside from IL-6R antagonists and JAK inhibitors, whether better targeted drugs exist requires more clinical data and pathogenesis studies.

## Conclusions

We reported the first case of tocilizumab therapy in immune dysregulation disorders caused by a STAT3 GOF mutation in mainland China. Gene sequencing should be performed for patients with early-onset refractory immune dysregulation diseases since routine immunological examination may be nonspecific. Targeted drugs can effectively improve the clinical problems associated with STAT3 gain-of-function mutations, while nontargeted immunosuppressive therapy is usually insufficient.

## Methods

The study was carried out at Children’s hospital of Fudan University (Shanghai, China). The protocol 2020–453 was approved by the Ethics Committee of the Children’s Hospital of Fudan University. Because the patient was under the age of 18 years, her parents gave written informed consent to participate tocilizumab therapy and report data in accordance with the declaration of Helsinki.

### Clinical data

We retrospectively summarized the clinical data of the STAT3 GOF patient.

### Whole-exome sequencing

WES and analysis protocols were adapted for genetic analysis. After obtaining the informed consent from the parents, genomic DNA samples were extracted from the whole blood of the patient and her parents with the QIAamp® DNA Blood Mini Kit (Qiagen, Hilden, Germany). The concentration and quantity of the DNA samples were measured using a NanoDrop ultraviolet spectrophotometer (Thermo Fisher Scientific, Waltham, MA, USA) [19]. Genomic DNA fragments were enriched for the target region of the consensus coding sequence exons and sequenced on an Illumina HiSeq 2000 platform (Illumina, San Diego, CA) [19]. Data analyses were conducted by using ANNOVAR and VEP software in our clinical genetic laboratory by a bioinformatics team. The mutation was confirmed by Sanger sequencing.

### Routine evaluation of immunological function

Immunological evaluations were carried out during routine clinical work. As previously reported, IgG, IgA, and IgM were detected by nephelometry; IgE was assessed by UniCAP (Pharmacia, Uppsala, Sweden) [20]. Lymphocyte subsets were analyzed by FACSCalibur flow cytometer (Becton Dickinson, Franklin Lakes, NJ, USA) [21]. Whole blood was used for staining of lymphocyte surface markers and analyzed according to a standard multicolor flow cytometric protocol with following antibodies [21]: anti-CD3 (UCHT1), anti-CD8 (RPA-T8), anti-CD27 (M-T271), anti-CD45RA (HI100), anti-CD4 (RPA-T4), anti-TCRαβ (T10B9.1A-31), anti-TCRγδ (B1), anti-CD19 (HIB19), anti-CD24 (ML5), anti-CD38 (HIT2), anti-IgD (IA6–2), and anti-CD57 (NK-1) (all from BD Biosciences).

## Supplementary Information

**Additional file 1: Supplement Table 1.** Variants identified by WES.

**Additional file 2: Supplement Table 2.** OGTT results of our case (P1).

## Data Availability

The datasets used and analyzed during the current study available from the corresponding author on reasonable request.

## Abbreviations

STAT3: Signal transduction and activator of transcription 3
JAK: Janus kinases
LOF: Loss-of-function
GOF: Gain-of-function
NGS: Next generation sequencing
IUIS: International union of immunological societies
HLH: Lymphohistiocytosis syndrome
EBV: Epstein-barr virus
EM: Effector memory
IL-6CM: Central memory
IVIG: Intravenous immunoglobulin
