# Supplementary material for: Efficacy of tocilizumab therapy in a patient with severe pancytopenia associated with a STAT3 gain-of-function mutation
Source: BMC Immunol. 2021 Mar 17;22:19. doi: 10.1186/s12865-021-00411-1 (PMC7968248; doi:10.1186/s12865-021-00411-1)
Supplement: Supplementary file 1 — Additional file 1: Supplement Table 1. Variants identified by WES. [file 12865_2021_411_MOESM1_ESM.docx]

**Supplement Table 1 Variants identified by WES**

| **Inheritance mode** | **Gene** | **Variant** | **Allele origin** | **Polyphen** | **Sift** | **Mutation**  **Tester** | **CADD score** | |
| --- | --- | --- | --- | --- | --- | --- | --- | --- |
| **AD** | **STAT3** | **NM_139276: exon14: c.1261G>A (p.G421R)** | **de novo** | **D** | **D** | **D** | | **33** |
| AD/AR | CFTR | NM_000492: exon4:  c.374T>C (p.I125T) | Maternal | B | D | D | | 23.1 |
| AR | LRBA | NM_006726: exon41:  c.6363+5009C>T | Maternal | B | T | D | | 22.5 |
| AD | KMT2D | NM_003482: exon8:  c.1043G>A (p.R348H) | Paternal | B | T | N | | 21.6 |
| AR | UNC13D | NM_199242: exon14:  c.1280G>A (p.R427Q) | . | D | D | D | | 31 |
| AR | RNASEH2A | NM_006397: exon4:  c.340A>G (p.N114D) | . | D | D | D | | 27.4 |
| AD/AR | POLE | NM_006231: exon46:  c.6475C>T (p.R2159C) | Paternal | B | D | D | | 25.2 |
| AD/AR | POLE | NM_006231: exon14:  c.1471G>A (p.E491K) | . | D | D | D | | 27.8 |
| AD/AR | FOXN1 | NM_003593: exon3:  c.689C>G (p.P230R) | . | B | T | N | | 24.0 |
| AR | TMC6 | NM_007267: exon5:  c.385C>T (p.R129C) | . | B | D | D | | 23.4 |

NOTE. Inheritance mode: AD autosomal dominant, AR autosomal recessive.

Polyphen: D represent probably damage with polyphen score 0.957~1, B represents benign with score 0~0.452

SIFT: D represent Deleterious with SIFT score<0.05, T represent Tolerate with score>0.05.

Mutation Taster: D represents disease causing, N represents polymorphism.
