# Supplementary material for: Efficacy of tocilizumab therapy in a patient with severe pancytopenia associated with a STAT3 gain-of-function mutation
Source: BMC Immunol. 2021 Mar 17;22:19. doi: 10.1186/s12865-021-00411-1 (PMC7968248; doi:10.1186/s12865-021-00411-1)
Supplement: Supplementary file 2 — Additional file 2: Supplement Table 2. OGTT results of our case (P1). [file 12865_2021_411_MOESM2_ESM.docx]

**Supplement Table 2 OGTT results of our case (P1)**

| Time  (minutes) | C peptide  (ng/ml) | Plasma insulin  (pmol/L) | Plasma glucose  (mmol/L) |
| --- | --- | --- | --- |
| 0 | 3.60 | 50.7 | 8.1 |
| 30 | 8.68 | 110.4 | 16.6 |
| 60 | 8.86 | 89.3 | 13.9 |
| 120 | 12.2 | 60.9 | 13.3 |
| 180 | 12.2 | 139.6 | 11.5 |
